# Genomic characterization of the first goat-associated orf virus genome sequenced directly from a human clinical sample in Portugal

**DOI:** 10.1007/s00705-026-06749-4

**Published:** 2026-09-28

**Authors:** Daniel Sobral, Rita Cordeiro, Ana Paula Brito, Ana Margarida Henriques, Sílvia C. Barros, Fábio Abade dos Santos, Margarida D. Duarte, Ana Pelerito, Isabel Lopes de Carvalho, Luís Coelho, Rita Ferreira, Sara Rangel, Luís Vieira, Maria Sofia Núncio, Vítor Borges, João Paulo Gomes

**Affiliations:** 1 Genomics and Bioinformatics Unit, Department of Infectious Diseases, National Institute of Health Doutor Ricardo Jorge (INSA), Lisbon, Portugal; 2 Emergency Response and Biopreparedness Unit, Infectious Diseases Department, National Institute of Health Doutor Ricardo Jorge (INSA), Lisbon, Portugal; 3 Environment & Infectious Diseases Research Group, Institute of Environmental Health (ISAMB), Faculty of Medicine, University of Lisbon, Lisbon, Portugal; 4 Service of Infectious Diseases, Local Health Unit Almada–Seixal (ULSAS), Almada, Portugal; 5 Laboratory of Virology, Strategic Unit for Animal Production Research and Services, National Institute for Agrarian and Veterinary Research, Av da República, Quinta do Marquês, Oeiras, 2780-157 Portugal; 6 Department of Life Sciences, NOVA School of Science and Technology, Caparica, Portugal; 7 Veterinary and Animal Research Centre (CECAV), Faculty of Veterinary Medicine, Lusófona University, Lisbon, Portugal; 8 Associate Laboratory for Animal and Veterinary Sciences (AL4AnimalS), Avenida da Universidade de Lisboa, Lisboa, 1300-477 Portugal; 9 Technology and Innovation Unit, Department of Human Genetics, National Institute of Health Doutor Ricardo Jorge (INSA), Lisbon, Portugal

## Abstract

The orf virus (ORFV) is a neglected zoonotic virus, with sporadic human infections, historically identified through clinical suspicion rather than molecular confirmation. We report the first molecularly confirmed human ORFV case in Portugal and the first goat-associated human-derived ORFV genome. Direct hybrid capture sequencing of a clinical biopsy from a veterinary student bitten by a goat yielded a 129.5 kb genome. Phylogenetic analysis placed the isolate within a goat-associated cluster, distinct from the two previously available human-derived genomes. This case highlights the value of molecular surveillance and genomic sequencing for One Health surveillance of neglected zoonoses.

**Supplementary Information:**

The online version contains supplementary material available at https://doi.org/10.1007/s00705-026-06749-4.

## Introduction

The orf virus (ORFV) is a zoonotic, epitheliotropic virus belonging to the *Parapoxvirus* genus (family *Poxviridae*), causing contagious ecthyma in sheep and goats with occasional human cases predominantly associated with occupational hazards [1]. Its genome is approximately 130–140 kbp with an unusually high G + C content (63–65%). Although well recognized in livestock, the human burden of ORFV is probably underestimated since infections are not notifiable in many countries and dedicated surveillance systems are lacking [2], leaving the true disease burden uncertain.

Human infection typically results from direct inoculation of abraded skin through contact with infected animals or contaminated materials [1, 3]. Human-to-human transmission is exceptionally rare, with only a few documented cases [4]. Despite a characteristic lesion evolution, the clinical presentation can resemble other vesiculopustular zoonoses [1, 3], and in routine practice, diagnosis is often based on clinical grounds and exposure history alone. Molecular confirmation by PCR targeting conserved genes such as *B2L* provides definitive diagnosis and differentiates ORFV from other poxviruses [5]. The few published human cases originated from isolated clinical reports rather than coordinated molecular surveillance programs [6–9]. At the genomic level, ORFV remains underrepresented in public databases, with only two complete human-derived ORFV genomes (IHUMI-1 and B029) publicly accessible [8, 10].

One major limitation in ORFV genomic characterization has been the difficulty of isolating and propagating it in standard laboratory cell lines [8]. However, recent advances in molecular methodologies, including multi-viral hybrid capture panels such as the Comprehensive Viral Research Panel (CVRP, Twist Bioscience) and the Viral Surveillance Panel (VSP, Illumina), now enable viral whole-genome data to be captured directly from clinical samples. These approaches substantially reduce reliance on viral culture and open new possibilities for systematic genomic surveillance.

In Portugal, three cases of ORFV infection in humans have been reported over the past decade, all clinically diagnosed but lacking laboratory confirmation [11–13]. Sporadic ORFV cases in sheep have also been confirmed by molecular testing at INIAV. The present study reports the first laboratory-confirmed human ORFV case in Portugal and provides the genome sequence of the identified strain, thereby contributing to the molecular epidemiology of this neglected zoonotic virus.

## Materials and methods

**Fig. 1:**
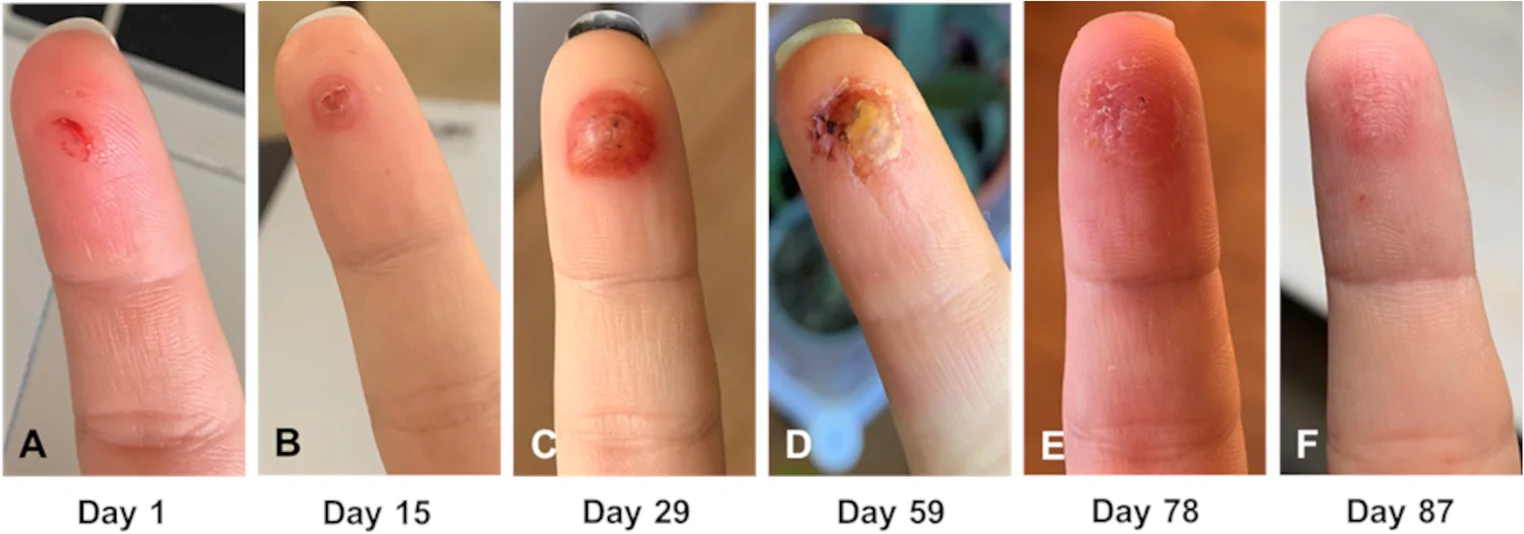
Temporal evolution of a finger lesion following a goat bite. (**A**) Day 1. Initial lesion on the finger immediately after a goat bite. (**B**) Day 15. After careful wound cleaning and disinfection, a small whitish nodule surrounded by an erythematous halo appeared. The lesion was punctured due to suspicion of purulent content; however, no material was expressed. (**C**,** D**) Days 29–59. The lesion became reddish with dark red and black spots, developing a firm capsule-like structure. It remained sensitive and painful on pressure. A biopsy was performed on day 44, and sutures were removed on day 57. (**E**, **F**) Day > 78. Healing stage. The skin remained slightly sensitive and not fully smooth, with a faint circular mark at the site of the previous lesion

A cutaneous biopsy was obtained from a 23-year-old female veterinary medicine student presenting with a 5 mm solitary erythematous nodule on her right-hand finger (Fig. 1), approximately four weeks after being bitten by a goat reported to have visible oral lesions (though specific diagnosis of the animal was not performed). The initial histopathological examination suggested chondroma, inconsistent with the clinical presentation and epidemiological history, prompting molecular investigation at INSA. DNA was extracted using the QIAamp DNA Mini Kit (Qiagen). *Parapoxvirus* detection was performed using an in-house real-time PCR targeting the *B2L* gene, as previously described [14], and specific ORFV detection was subsequently confirmed using a commercial assay (Biopremier), both run on the CFX Opus Real-Time PCR System (Bio-Rad).

Sequencing libraries were prepared with the Twist Total Nucleic Acids Library Preparation EF Kit 2.0, starting with fragmentation (8 min at 30 °C) to optimize insert size. Ligation of Twist Universal Adapters was followed by 12-cycle amplification with Twist UDI Primers (Twist Bioscience). Quality control was performed using the Qubit dsDNA Broad Range Quantitation Assay (Thermo Fisher Scientific) and the Fragment Analyzer (Advanced Analytical Technologies). Indexed libraries were pooled and hybridized with the Twist Comprehensive Viral Research Panel (~ 16 h at 70 °C). Captured libraries were amplified (8 cycles), purified, and normalized to 2 nM, with a final pool quantified on a Qubit 3.0 fluorometer. Paired-end sequencing was performed on an Illumina MiSeq (2 × 250-bp).

Viral screening and reference-based consensus assembly were performed in INSaFLU [15] version 2.2.2 (TELEVIR and consensus modules). Illumina reads were quality-processed with FastQC and Trimmomatic. Quality-processed reads were assembled with SPAdes to generate draft contigs, which were blasted against the NCBI core database. The most similar complete ORFV genome (OR637323.1) was used for reference-based consensus generation in INSaFLU: quality-filtered reads were mapped with Snippy against reference OR637323.1, and a majority consensus was obtained for positions with coverage greater than 10x (missing or lower coverage positions were marked as ‘N’). Reads were remapped against the consensus and manually inspected for artifacts in the Integrative Genome Viewer. Genome annotations from the OV-SA-00 reference strain were transferred to the final consensus using the Genome Annotation Transfer Utility and annotation comparisons were visualized with clinker.

For phylogenetic context, all 57 complete ORFV genomes available in NCBI (accessed 18 February 2026) were included, along with a newly generated genome from an ORFV-positive sheep sample (unrelated to the clinical case) collected in 2024 at INIAV (ORFV/Ov/PT001/2024; Alentejo, Portugal). For this new sample, nucleic acids from scabs were extracted using the IndiMag Pathogen Kit (Indical Bioscience) on a KingFisher Flex instrument (ThermoFisher Scientific). Parapoxvirus detection was performed as previously described [16], and sequencing/assembly used the same workflow described above. The ORFV genomes were aligned with MAFFT (v7.526, parameter “--auto”) and uninformative loci were filtered using TrimAl (v1.2, parameter “-automated1”). Whole-genome phylogeny was reconstructed with IQ-TREE (version 3.0.1, parameters “-m MFP -bb 1000 -alrt 1000 -o GQ329670.1”) from the filtered MAFFT alignment, using Pseudocowpox virus (GQ329670) as outgroup. For the B2L-specific analysis, all complete ORFV B2L sequences available in NCBI were downloaded, along with their associated metadata (partial B2L sequences were not considered). We also extracted complete B2L gene sequences from whole genomes, achieving a total of 353 complete B2L sequences. A *B2L*-specific phylogeny was built with a Nextstrain [17] generic build in INSaFLU, (https://github.com/INSaFLU/nextstrain_builds/tree/main/generic). Briefly, sequences were aligned using MAFFT and a tree inferred using IQ-TREE (default parameters, without bootstrap). The maximum likelihood (ML) distance matrix from IQ-TREE was used to perform a Principal Coordinates Analysis (PCoA) in python (*n* = 297 annotated sequences associated either to goat *n* = 203, sheep *n* = 87 or human *n* = 7). For the host-association analysis (only goat and sheep were considered), host information was randomly shuffled 1000 times, and the mean ML distance between samples with the same host was compared to the mean distance in the true matrix.

## Results

**Fig. 2:**
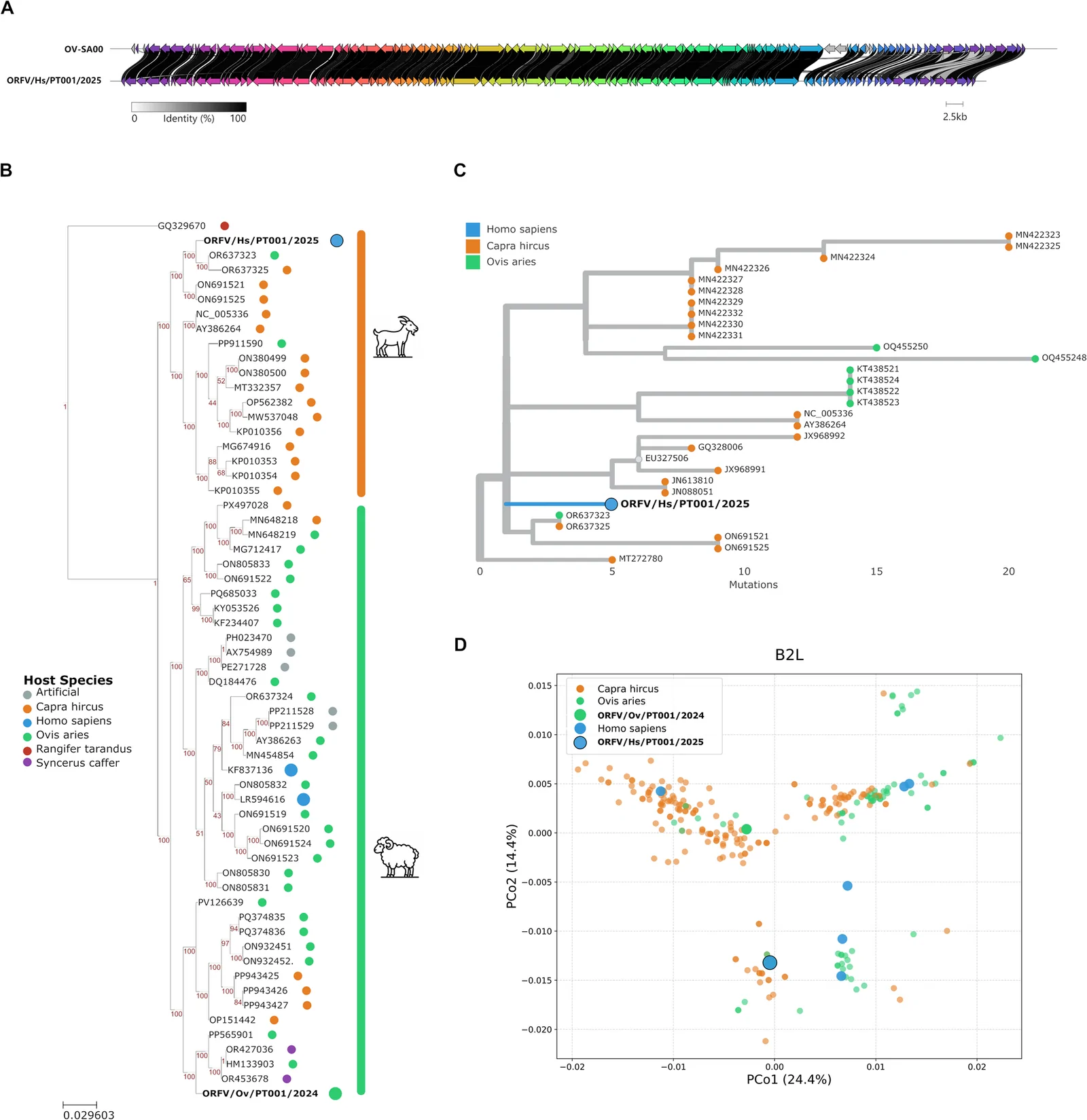
Phylogenetic characterization of the sequenced genome. (**A**) Comparison between the genomes of OV-SA00 (AY386264) and ORFV/Hs/PT001/2025 (this study). (**B**) Whole-genome IQ-TREE phylogenetic tree of 57 publicly available ORFV consensus sequences, plus two newly sequenced genomes from Portugal (in bold). Branch support values represent 1000 bootstrap replicates. Colors indicate host species, with the human-derived Portuguese clinical isolate highlighted in the goat-associated clade, distinct from the sheep-associated human-derived isolates IHUMI-1 and B029. (**C**) Zoomed Nextstrain phylogenetic tree of the B2L gene indicating the nucleotide differences between the Portuguese human-derived isolate and its closest relatives. (**D**) Principal Coordinate Analysis (PCoA) of 297 ORFV isolates suggesting genomic segregation by host species (*Capra hircus* vs. *Ovis aries*). Permutation testing suggests significant host-association (*p* < 0.001). Human-derived isolates are highlighted

Molecular analysis of the human biopsy specimen revealed the presence of *Parapoxvirus* DNA, confirming a diagnosis of human ORFV infection and overturning the initial misclassification based on histopathology. Direct sequencing of the human clinical sample yielded 2,350,114 reads, approximately 16% of which were non-human. Over 98% (363,734 reads) of these were of ORFV sequences. A consensus sequence (ORFV/Hs/PT001/2025) covering 99.6% of the OR637323 reference genome was obtained with an average coverage of 1000x, spanning 129.5 kb with a 64.6% GC content. Of the 130 annotated genes from the OV-SA-00 reference strain, 117 were detected at greater than 90% similarity (110 at greater than 95% similarity - Fig. 2A, Supp. Table 1). Genes commonly utilized for phylogenetic analysis, including *B2L* (ORF011), F1L (ORF059), and VIR (ORF020) were completely assembled.

A genome-wide phylogenetic tree was constructed to compare this ORFV genome with other publicly available ORFV genomes (Fig. 2B). The Portuguese strain associated with the human clinical case (ORFV/Hs/PT001/2025) had the highest similarity to sequences in Cuba (OR637323 and OR637325) and Italy (ON691521 and ON691525), grouping mostly with goat-associated samples. Conversely, the two other complete genomes available for human-associated ORFV (LR594616/IHUMI-1 and KF837136/B029) clustered more closely with sheep-associated strains.

Phylogenetic studies of ORFV usually rely on individual genes, such as *B2L* [18], for which more data is available. In order to expand on the limited whole-genome dataset, a *B2L*-specific phylogenetic tree was constructed using 363 complete *B2L* gene sequences (Fig. 2C). The *B2L* sequence of the human-derived Portuguese strain differs from the aforementioned Cuban samples by only six base pairs, which remain the most similar isolates. A Principal Coordinates Analysis (PCoA) based on the *B2L* distance matrix of 297 sequences from goat, sheep, and human hosts (Fig. 2D) confirmed the proximity of the Portuguese strain to other goat-associated samples, and suggested some degree of host-preference (*p* < 0.001, based on 1000 randomly shuffled distance matrices), although frequent intermingling of host species was also observed. Moreover, although the first axis of the PCoA seems to capture host-specificity, there is still much variance to be explained, suggesting a complex and multidimensional genetic diversity even when only considering the *B2L* gene.

Of note, we have also obtained an ORFV genome (370-fold mean coverage) directly from a sheep sample collected in 2024 and diagnosed at INIAV (ORFV/Ov/PT001/2024). This genome showed marked divergence from the ORFV genome recovered from the 2025 human case, supporting the circulation of distinct lineages in Portugal. Phylogenetically, the sheep-derived genome clustered more closely with the D1701 reference (PP565901) within the “sheep-associated” branch (Fig. 2B).

## Discussion

To our knowledge, this case represents the first molecularly confirmed report of human ORFV infection in Portugal. The case illustrates the typical presentation of human ORFV infection, namely a solitary cutaneous lesion occurring in an individual with occupational exposure risk [1] following direct contact with an infected animal. However, the clinical course and diagnostic trajectory of this sample also highlights a critical challenge in clinical virology, namely, the potential for histopathological misinterpretation when viral cytopathic changes are subtle or when the biopsy specimen fails to include representative areas of infection. The initial misclassification as a chondroma — a highly unusual diagnosis considering both the anatomical location and clinical and epidemiological context — underscores the decisive value of molecular testing, including real-time PCR and next-generation sequencing, in establishing definitive diagnoses when clinical history strongly suggests a zoonotic orf infection. This observation underscores the need for improved clinical awareness and better integration of epidemiological data into diagnostic workflows.

Historically, genomic characterization of ORFV has been hindered by the difficulty of culturing the virus in vitro [8]. In this work, a comprehensive viral capture panel was used to successfully perform high-depth whole-genome sequencing of ORFV directly from the biopsy tissue, thus avoiding the biases and limitations of viral culture. The resulting 129.5 kb genome is the third ORFV genome of human origin to be made publicly available worldwide. Although it was not possible to obtain a sample from the suspected animal to directly demonstrate the zoonotic event (unlike the case of the human-derived B029 strain [10]), whole genome phylogenetic analyses placed the sequence within a branch with samples mostly derived from goats, in agreement with the patient’s self-report of being bitten by a goat that presented lesions in the mouth area. This contrasts with the two previously published complete genomes of human origin (from France [8] and Germany [10]), which show closer phylogenetic relationships with other sheep-associated strains. Therefore, the genomic composition of the human-derived Portuguese strain provides complementary data to the existing databases, representing genetic diversity that has not been previously sampled in human infections.

Despite the potential host-association phylogenetic signal (Fig. 2B) [19], frequent cross-species circulation of ORFV lineages is observed, as exemplified by the two sequences from Cuba that were found to be the most similar to the ORFV/Hs/PT001/2025, which were collected from different hosts (sheep and goat). Furthermore, an analysis using an expanded set of complete *B2L* gene sequences supports the results of the whole-genome phylogeny. While the *B2L* distance matrix and PCoA analysis suggests some degree of host-preference among ORFV strains, sampling bias may be a confounding factor. Moreover, the frequent intermingling suggests broad inter-species transmission capabilities.

Besides the case reported here, a few cases of possible ORFV infection have been detected in Portugal [11–13]. Combined with sporadic laboratory-confirmed detections by INIAV, this suggests that the virus may be widely distributed among small ruminant populations. Still, there is virtually no information on the genetic diversity of ORFV circulating in the country, and only limited data is available for the Iberian Peninsula [19, 20]. Although the single animal ORFV genome from 2024 we obtained in this study is a limited contribution, it suggests that a wide, unsampled ORFV diversity is present in Portugal.

Given the importance of sheep and goat farming in many Portuguese regions, and the fact that human ORFV infections are usually self-limiting and mild and can resemble other dermatological conditions, there is a risk of misdiagnosis or underreporting in clinical settings. Therefore, occupational exposure to this virus among farmers and veterinarians could be an overlooked source of zoonotic infection. Enhanced molecular surveillance is therefore critical for resolving atypical clinical presentations. Combined with the application of culture-independent whole-genome sequencing methods, it could significantly contribute towards an improved genomic characterization of this virus, a broader understanding of cross-species transmission events, and allow accurate mapping of the evolutionary history of this neglected zoonotic pathogen. This case further underscores the importance of adopting a One Health approach, reinforcing the need for a closer collaboration between the veterinary and human health sectors to improve diagnosis capacity and enhance the understanding and surveillance of this neglected zoonosis.

In summary, this study documents the first laboratory-confirmed human ORFV infection in Portugal, directly sequenced from a clinical sample. In combination with epidemiological data, the resulting genome suggests zoonotic transmission of a goat-associated ORFV lineage in Portugal. It also provides the third publicly accessible ORFV genome sequence of clinical origin and the first human-derived ORFV genome primarily associated with goats.

## Supplementary Information

Below is the link to the electronic supplementary material.

Supplementary Material 1 (XLSX 14.9 KB)

Supplementary Material 2 (ZIP 43.1 KB)

## Data Availability

The assembled ORFV consensus sequences are deposited in NCBI (accessions PZ497123 and PZ497124). Sequence reads aligning against the generated ORFV consensus sequences are deposited in the European Nucleotide Archive (ENA) under the BioProject PRJEB114135. The whole-genome consensus and B2L Nexstrain phylogenies (which can be visualized with tools such as auspice), as well as accompanying metadata are provided as Supplemental Data.
